# Autoimmune Neurologic Disorders

**DOI:** 10.2174/157015911796557993

**Published:** 2011-09

**Authors:** Saša Živković

Autoimmune disorders affect 5-10% of the general population, [1] and often involve central and peripheral nervous system. In this special issue of *Current Neuropharmacology* experts discuss pathophysiology, clinical features and treatment of various neurologic autoimmune disorders. In the last decade we have witnessed remarkable scientific advances which have greatly improved our understanding of pathophysiology of the autoimmune disorders leading to an increasing number of treatment options with many of new therapies already being used in the treatment of neurologic disorders as their role continues to evolve [2]. The first article summarizes pathophysiology, diagnosis and treatment of neuromuscular autoimmune conditions including Guillain Barre syndrome, myasthenia gravis and polymyositis. This is followed by a comprehensive review of treatment of multiple sclerosis by Drs. Loma and Heyman describing standard treatment protocols as well as newly approved treatments. The next article by Drs. Awad and Stuve highlights clinical features and pathophysiology of idiopathic transverse myelitis and neuromyelitis optica which often result in significant disability. Dr. David Lacomis discusses clinical features and treatment of neurosarcoidosis which may affect central and/or peripheral nervous system, usually in combination with multisystemic disease. Dr. Maria Cid and her colleagues provide a detailed review of systemic and primary CNS vasculitides and their pathophysiology and treatment. Neuropsychiatric lupus is often called a “great mimicker” due to its wide spectrum of clinical manifestations, and Drs. Kao and Popescu review its pathophysiology and treatment. Over the last few years, autoimmune channelopathies have become one of the hot topics of basic and clinical research, and Dr. Kleopa reviews paraneoplastic and non-paraneoplastic autoimmune channelopathies. The issue concludes with a comprehensive review of toxicities of immunosuppressive treatment by Drs. Fadul and Lallana. Authors discuss potential risks of various therapies used in the treatment of autoimmune neurologic disorders.

I would like to thank the authors of this *Current Neuropharmacology* issue for all their time and effort in preparing their articles to create a comprehensive and up-to-date collection of articles on the wide spectrum of autoimmune neurologic disorders.
